# Delayed multiligament PCL reconstruction is associated with a higher prevalence of intraarticular injury and may influence treatment

**DOI:** 10.1186/s12891-023-06638-w

**Published:** 2023-06-19

**Authors:** Bálint Zsidai, Ian D. Engler, Eric Narup, Ryan T. Lin, Ehab M. Nazzal, Philipp W. Winkler, Kristian Samuelsson, James J. Irrgang, Volker Musahl

**Affiliations:** 1 Department of Orthopaedic Surgery, UPMC Freddie Fu Sports Medicine Center, University of Pittsburgh, Pittsburgh, USA; 2 Sahlgrenska Sports Medicine Center, Gothenburg, Sweden; 3 Department of Orthopaedics, Institute of Clinical Sciences, Sahlgrenska Academy, University of Gothenburg, Gothenburg, Sweden; 4 Central Maine Healthcare Orthopedics, Central Maine Medical Center, Auburn, USA; 5 Department of Orthopaedics and Traumatology, Kepler University Hospital Linz, Linz, Austria; 6 Department of Sports Orthopaedics, Klinikum Rechts Der Isar, Technical University of Munich, Munich, Germany; 7 Department of Orthopaedics, Sahlgrenska University Hospital, Mölndal, Sweden; 8 Department of Physical Therapy, School of Health and Rehabilitation Sciences, University of Pittsburgh, Pittsburgh, USA

**Keywords:** Posterior cruciate ligament, PCL, Surgical timing, Multiligament, Knee injury, Meniscus tear, Cartilage injury, Knee ligament

## Abstract

**Background:**

The aim of this study was to investigate differences in concomitant injury patterns and their treatment in patients undergoing early (≤ 12 weeks) and delayed (> 12 weeks) primary multiligament posterior cruciate ligament (PCL) reconstruction (PCL-R).

**Methods:**

This study was a retrospective chart review of patients undergoing primary multiligament PCL-R at a single institution between 2008 and 2020. Multiligament PCL-R was defined as PCL-R and concurrent surgical treatment of one or more additional knee ligament(s). Exclusion criteria included isolated PCL-R, PCL repair, and missing data for any variable. Patients were dichotomized into early (≤ 12 weeks) and delayed (> 12 weeks) PCL-R groups based on the time elapsed between injury and surgery. Between-group comparison of variables were conducted with the Chi-square, Fisher’s exact, and independent samples t-tests.

**Results:**

A total of 148 patients were eligible for analysis, with 57 (38.5%) patients in the early and 91 (61.1%) patients in the delayed multiligament PCL-R groups. Concomitant LCL/PLC reconstruction (LCL-R/PLC-R) was performed in 55 (60%) of delayed multiligament PCL-Rs and 23 (40%) of early PCL-Rs (*p* = 0.02). Despite similar rates of meniscus injury, concomitant meniscus surgery was significantly more prevalent in the early (*n* = 25, 44%) versus delayed (*n* = 19, 21%) multiligament PCL-R group (*p* = 0.003), with a significantly greater proportion of medial meniscus surgeries performed in the early (*n* = 16, 28%) compared to delayed (*n* = 13, 14%) PCL-R group (*p* = 0.04). The prevalence of knee cartilage injury was significantly different between the early (*n* = 12, 24%) and delayed (*n* = 41, 46%) multiligament PCL-R groups (*p* = 0.01), with more frequent involvement of the lateral (*n* = 17, 19% vs. *n* = 3, 5%, respectively; *p* = 0.04) and medial (*n* = 31, 34% vs. *n* = 6, 11%, respectively; *p* = 0.005) femoral condyles in the delayed compared to the early PCL-R group.

**Conclusions:**

Given higher rates of chondral pathology and medial meniscus surgery seen in delayed multiligament PCL-R, early management of PCL-based multiligament knee injury is recommended to restore knee stability and potentially prevent the development of further intraarticular injury.

**Level of evidence:**

Level III.

## Background

Posterior cruciate ligament (PCL) injury can be present in isolation or, commonly, as a component of a multiligamentous knee injury (MLKI) [1, 2]. While management of isolated PCL injuries is often nonoperative, PCL reconstruction is more commonly performed in the setting of MLKI [1–4]. Concomitant meniscal and chondral pathology is frequently observed in patients with PCL-based MLKI [2], consistent with the frequent mechanism of high-velocity, direct trauma to the knee [5]. Additionally, the high prevalence of intraarticular injury in the multiligament PCL-R population may related to the detrimental long-term effect of persistent anteroposterior and rotatory knee laxity on knee kinematics due to PCL deficiency [6–8]. While evidence is abundant for the impact of surgical timing in the setting of multiligament anterior cruciate ligament reconstruction [9], there is a paucity of literature on surgical timing in multiligament PCL-R [10–14]. Furthermore, existing studies have heterogeneous definitions of early and delayed surgery, which hinders conclusions about the effect of surgical timing on outcomes. Proposed advantages of early surgery include a reduced risk of further meniscus injury and cartilage lesions, earlier return to sport, the possibility of concomitant ligament repair, rapid treatment of concomitant intraarticular pathology, and reduced muscle atrophy [9]. In contrast, delayed surgery may improve preoperative knee range of motion and decrease the risk of arthrofibrosis and postoperative knee stiffness [9].

The purpose of this study was to investigate the impact of surgical timing on the prevalence of concomitant intraarticular and ligamentous injury in patients undergoing primary multiligament PCL-R. Early surgery was defined as ≤ 12 weeks, and delayed surgery was defined as > 12 weeks of time elapsed from injury to surgery [15]. It was hypothesized that delayed surgical treatment would be associated with increased meniscus and cartilage pathology, especially in the medial compartment due to a persistent increase in medial joint contact forces with PCL-deficiency [6, 7].

## Materials and methods

This retrospective cohort study was performed at a single academic center, with approval obtained from the University of Pittsburgh Institutional Review Board (STUDY20070271). All patient data used for this study was anonymized prior to analysis. The requirement for informed consent was waived under the approved University of Pittsburgh Institutional Review Board application. Methods of the current study were conducted in accordance with the Declaration of Helsinki. Patients undergoing primary multiligament PCL-R between January 1, 2008 and January 1, 2020 were eligible for inclusion. Multiligament PCL-R was defined as surgical reconstruction of the PCL concomitant with surgery to at least one other knee ligament, including anterior cruciate ligament reconstruction (ACL-R), lateral collateral ligament/posterolateral corner reconstruction (LCL-R/PLC-R), medial collateral ligament/posteromedial corner reconstruction (MCL-R/PMC-R), medial collateral ligament (MCL) repair, and posterolateral corner (PLC) repair. Patients with isolated PCL-R, revision PCL-R, PCL repair, and insufficient data were excluded from further analysis.

Patients were grouped according to the timing of multiligament PCL-R surgery, in which the early group included patients who underwent surgery within 12 weeks or less following injury (≤ 12 weeks), and the delayed group included patients who underwent surgery after 12 weeks from injury. Injury diagnosis was based on a combination of clinical examination and imaging studies, including radiography and magnetic resonance imaging (MRI).

### Data collection and outcomes

Patient charts were reviewed to collect patient demographics, including patient age, body mass index (BMI), sex, time from injury to surgery, injury laterality, injury mechanism (sport, motor vehicle accident, fall, other), and previous ipsilateral knee surgery. Injury- and surgery-related variables included concomitant ligament surgery (ACL-R, LCL-R/PLC-R, MCL-R/PMC-R, MCL repair, PLC repair), concomitant meniscus injury (medial versus lateral meniscus), meniscus surgery (repair versus partial meniscectomy), concomitant cartilage injury (locations including patella, trochlea, lateral femoral condyle, medial femoral condyle, lateral tibial plateau, medial tibial plateau, unreported), and concomitant cartilage surgery. Surgeon decision for non-intervention to a meniscus injury mostly factored in the pattern and size and location of the meniscus tear, as well as evidence of healing since the injury.

The primary outcome was the comparison of intraarticular injury prevalence—i.e., meniscus and cartilage injury—between the early and delayed multiligament PCL-R groups. Secondary outcomes were the differences in the rates of ligament, meniscus and cartilage surgeries performed concomitant with multiligament PCL-R between the early and delayed surgery groups.

### Statistical analysis

Statistical analysis for this study was conducted with SAS (Version 9.4. Cary, NC: SAS Institute Inc.). Mean, standard deviation (SD), frequency, and proportion (%) were used to describe the study population and patient groups. Categorical variables were compared with Chi-square or Fisher exact tests, while continuous variables were compared with independent samples t-tests or Kruskal–Wallis tests, where appropriate. Significance was set at *p* < 0.05.

## Results

### Patient demographics

Of the 148 patients included in the final analysis, the early multiligament PCL-R group consisted of 57 (39%) patients, while the delayed group consisted of 91 (61%) patients. The mean time from injury to surgery was 6.0 ± 3.1 weeks in the early multiligament PCL-R group and 63.7 ± 116.1 weeks in the delayed PCL-R group. Comparing the early versus delayed groups, there were no significant differences in the mean patient age, BMI, sex, injury laterality, and the prevalence of previous surgery (Table 1). While there was no significant difference between groups in the injury mechanism, sports (*n* = 18, 32%) were the most prevalent cause of injury in the early surgery group, and motor vehicle accidents were most prevalent (*n* = 41, 45%) in the delayed surgery group.

**Table 1:** Comparison of demographics in patients undergoing early and delayed multiligament PCL-R

	**Early (≤ 12 weeks)**	**Delayed (> 12 weeks)**	***P* value**
	**(*n* = 57)**	**(*n* = 91)**	
**Age [years]**	31 ± 13.6	29.5 ± 13.1	0.51
**BMI [kg/m**^**2**^**]**	30.5 ± 7.0	30.2 ± 7.8	0.81
**Time from injury to surgery [weeks]**	6 ± 3.1	63.7 ± 116.1	< 0.001
**Sex, n (%)**			0.69
Male	40 (70)	61 (67)	
Female	17 (30)	30 (33)	
**Laterality [right], n (%)**	24 (42)	42 (46)	0.63
**Injury mechanism, n (%)**			0.12
Sports	18 (32)	24 (26)	
Motor vehicle accident	15 (26)	41 (45)	
Fall	15 (26)	18 (20)	
Other	9 (16)	8 (9)	
**Previous surgery [yes], n (%)**	17 (30)	40 (44)	0.09

Age, BMI, and time from injury to surgery are presented as mean ± SD. Values are presented as count (n) and proportion (%) for sex, laterality of injury, injury mechanisms and previous surgery. Between group differences were analyzed using independent samples t-tests and Chi-square or Fischer’s Exact tests. In both early and delayed PCL-R groups, the value for BMI is missing for 1 patient

Body mass index *PCL-R*  Posterior cruciate ligament reconstruction; *SD* Standard deviation

### Injury- and surgery-related variables

**Fig. 1:**
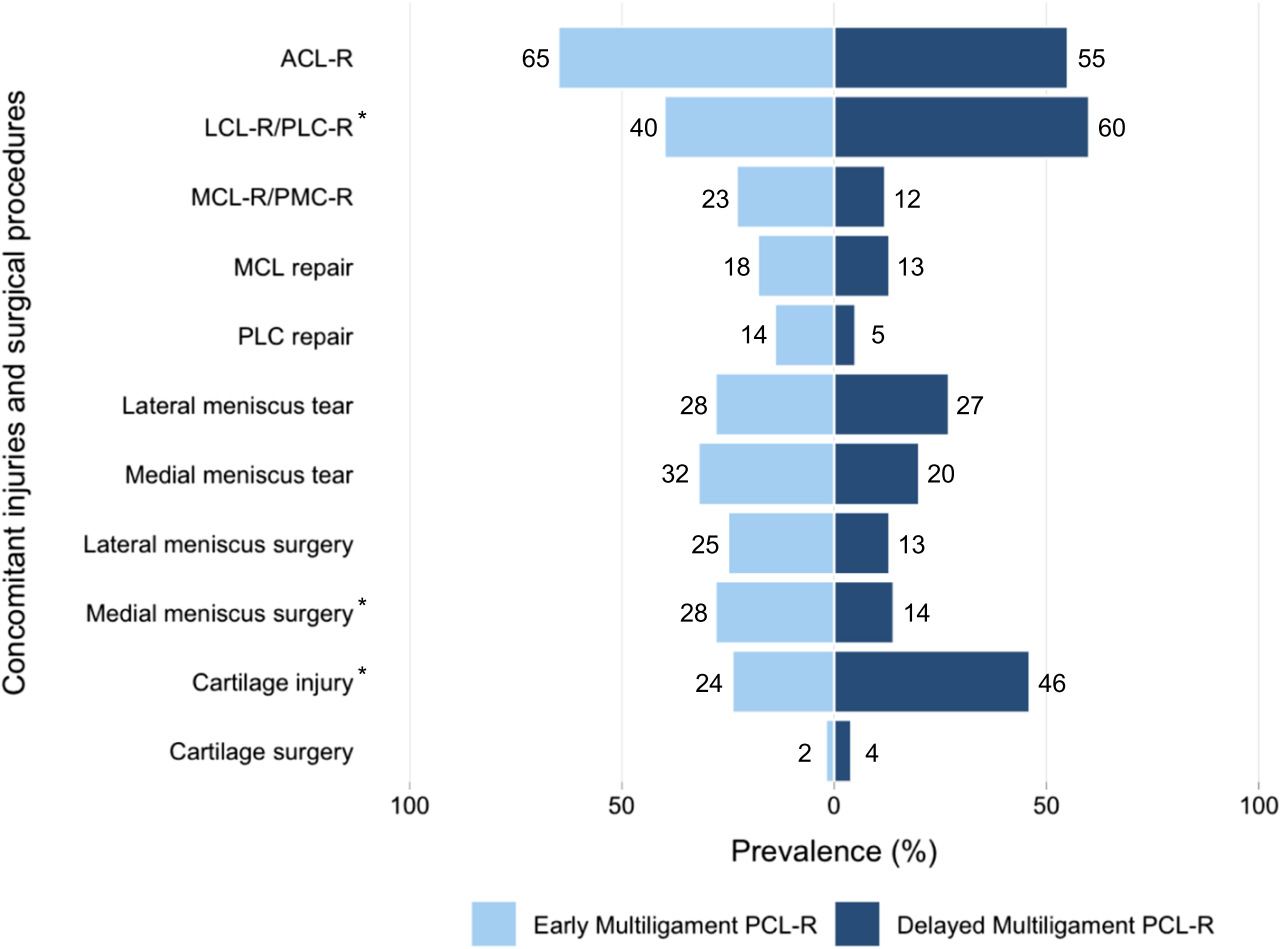
Deviating bar plot showing rates of concomitant injuries and surgical procedures in early and delayed multiligament PCL-R Asterisks (*) denote significant between-group differences at *p* < 0.05. ACL-R = anterior cruciate ligament reconstruction; LCL-R/PLC-R = lateral collateral ligament reconstruction/posterolateral corner reconstruction; MCL = medial collateral ligament; MCL-R/PMC-R = medial collateral ligament reconstruction/posteromedial corner reconstruction; PCL-R = posterior cruciate ligament reconstruction

There was a significantly greater prevalence of LCL-R/PLC-R in the delayed (*n* = 55, 60%) compared to the early (*n* = 23, 40%) multiligament PCL-R group (*p* = 0.02; Fig. 1). Otherwise, there was no significant between-group difference in the prevalence of specific concurrent ligament reconstruction or repair alongside the PCL-R (Table 2).

**Table 2:** Concomitant ligamentous and intraarticular injuries and their treatment in patients undergoing early and delayed multiligament PCL-R

	**Early (≤ 12 weeks)**	**Delayed (> 12 weeks)**	***P* value**
	**(*n* = 57)**	**(*n* = 91)**	
**Concomitant ligament surgery, n (%)**			
ACL-R	37 (65)	50 (55)	0.23
LCL-R/PLC-R	23 (40)	55 (60)	0.02*
MCL-R/PMC-R	13 (23)	11 (12)	0.09
MCL repair	10 (18)	12 (13)	0.47
PLC repair	8 (14)	5 (5)	0.07
**Concomitant meniscus injury, n (%)**			
Any meniscus tear	28 (49)	36 (40)	0.25
Lateral meniscus tear	16 (28)	25 (27)	0.94
Medial meniscus tear	18 (32)	18 (20)	0.1
**Any meniscus surgery, n (%)**	25 (44)	19 (21)	0.003*
**Concomitant lateral meniscus surgery, n (%)**	14 (25)	12 (13)	0.08
Partial meniscectomy	6 (43)	8 (67)	0.22
Repair	8 (57)	4 (33)	0.22
**Concomitant medial meniscus surgery**	16 (28)	13 (14)	0.04*
Partial meniscectomy	7 (44)	6 (46)	0.9
Repair	9 (56)	7 (54)	0.9
**Concomitant cartilage injury, n (%)**			
Any cartilage injury	12 (24)	41 (46)	0.01*
Patella	8 (14)	17 (19)	0.71
Trochlea	7 (12)	10 (11)	0.59
Lateral femoral condyle	3 (5)	17 (19)	0.04*
Medial femoral condyle	6 (11)	31 (34)	0.005*
Lateral tibial plateau	8 (14)	21 (23)	0.33
Medial tibial plateau	6 (11)	22 (24)	0.09
Unreported/missing	8 (14)	1 (1)	-
**Concomitant cartilage surgery, n (%)**	1 (2)	4 (4)	0.65

All values are presented as count (n) and proportion (%). Between group differences were analyzed using Chi-square or Fischer’s Exact tests. Cartilage injury was unreported/missing for 1 patient in the delayed PCL-R group (> 12 weeks) and 8 patients in the early PCL-R group (≤ 12 weeks). Asterisks (*) denote significant between-group differences at *p* < 0.05

*ACL-R* Anterior cruciate ligament reconstruction, *LCL-R* Lateral collateral ligament reconstruction, *MCL* Medial collateral ligament, *MCL-R* Medial collateral ligament reconstruction, *PLC* Posterolateral corner, *PLC-R* Posterolateral corner reconstruction, *PMC-R* Posteromedial corner reconstruction, *PCL-R* Posterior cruciate ligament reconstruction

Regarding concomitant meniscus injury, there was no significant difference in the overall prevalence of any meniscus tear between the early (*n* = 28, 49%) and delayed (*n* = 36, 40%) surgery groups (*p* = 0.25). The same was true when stratifying by tears in the medial meniscus and lateral meniscus separately (Table 2).

The overall rate of concomitant meniscus surgery was significantly greater in the early (*n* = 25, 44%) compared to the delayed (*n* = 19, 21%) surgery group (*p* = 0.003). When evaluating the medial meniscus alone, the rate of concomitant meniscus surgery was also significantly greater in early (*n* = 16, 28%) compared to delayed (*n* = 13, 14%) multiligament PCL-R patients (*p* = 0.04). There were no significant between-group differences in the isolated rates of partial meniscectomies or repairs (Table 2).

There was greater overall prevalence of concomitant cartilage injury in the delayed (*n* = 41, 46%) compared to the early (*n* = 12, 24%) multiligament PCL-R group (*p* = 0.01). The prevalence of cartilage injury was significantly greater on the lateral femoral condyle (*n* = 17, 19% vs. *n* = 3, 5%, respectively; *p* = 0.04) and the medial femoral condyle (*n* = 31, 34% vs. *n* = 6, 11%, respectively; *p* = 0.005) in delayed compared to early multiligament PCL-R patients. Other specific cartilage surfaces showed no significant between-group differences in the prevalence of chondral injury (Table 2). Concomitant cartilage injury status was unreported or missing in 8 (14%) patients in the early and 1 (1%) patient in the delayed multiligament PCL-R group. There was no significant difference in the rate of concomitant cartilage surgery with multiligament PCL-R between early (*n* = 1, 2%) and delayed (*n* = 4, 4%) surgery groups (*p* = 0.65).

## Discussion

The main findings of this study were the increased prevalence of cartilage injury, and the decreased rate of concomitant meniscus surgery despite similar rates of meniscus injury with delayed (> 12 weeks) versus early (≤ 12 weeks) multiligament PCL-R. These findings suggest that delaying the timing of multiligament PCL-R beyond 12 weeks may increase the susceptibility of patients to additional cartilage and meniscus injury prior to surgery, likely due to persistent knee instability and abnormal knee joint loading associated with sustained PCL-deficiency. Therefore, in patients with PCL-based MLKI, early surgical management may be important to restore knee stability, and potentially prevent further intraarticular pathology and the development of posttraumatic osteoarthritis [16].

### Intraarticular injuries and their treatment

Regarding intraarticular injuries, the current study identified a higher rate of meniscus surgery in patients with early compared to delayed multiligament PCL-R, which implies that early surgical timing may have a clinically significant impact on the feasibility of meniscus tear treatment in MLKI. The current consensus strongly encourages meniscus preservation as the first-line of treatment of reparable meniscus tears, with favorable long-term outcomes compared to meniscectomy [17]. Meniscal management is relevant to the MLKI population given that meniscus tears and chondral damage are frequent in patients with knee dislocations and MLKI [2, 10, 13, 18]. One study of 303 patients with knee dislocations reported a 37.3% prevalence of meniscus and 28.3% prevalence of cartilage injury, consistent with the high rate of intraarticular injury seen with MLKI in the current study. Furthermore, a fourfold greater odds of chondral injury was reported in patients who underwent surgical treatment in the chronic phase of injury (> 3 weeks) [10]. Another study of 122 patients with PCL-based MLKIs demonstrated a 55% prevalence of concurrent meniscus injury [18]. The same study found a 25% rate of bicompartmental or tricompartmental chondral damage in patients with delayed surgery (> 12 weeks) compared to only 6% in patients with early surgery (≤ 12 weeks) [18], in accordance with the greater rate of cartilage injury to both lateral and medial femoral condyles in the current investigation. Furthermore, inferior 6-year postoperative International Knee Documentation Committee (IKDC) scores were reported in patients with combined lateral and medial meniscus injury or chondral damage in the setting of knee dislocation [19]. Therefore, the importance of meniscus repair and early surgical treatment to avoid further intraarticular lesions should be emphasized in the multiligament PCL-R patient population.

### Prevalence and consequences of LCL and PLC injury

Injury to the PLC is one of the most frequently reported ligamentous injuries concomitant with multiligament PCL-R [1, 2]. The present study reported concomitant LCL-R/PLC-R in over half of patients with multiligament PCL-R, with a greater prevalence seen in the delayed surgery group. Combined PCL and PLC injuries result in increased contact pressures in the medial compartment of the knee [6], with an increased prevalence of chondral damage to the medial femoral condyle [20]. The current study is concordant with these findings, as the increased rate of LCL-R/PLC-R in the delayed multiligament PCL-R group was accompanied by a greater prevalence of injury to both the lateral and medial femoral condyles compared with the early surgery group. Consequently, surgeons should consider early treatment of MLKIs with combined PCL and LCL/PLC injury to avoid further risk of chondral injury due to prolonged anteroposterior and rotatory instability [8, 21].

### Biomechanical effect of persistent knee instability

Clinical findings of increased meniscal and chondral pathology in knees that have experienced sustained knee instability from delayed management of MLKI have biomechanical underpinnings. Ample literature highlights the negative impact of altered knee kinematics and joint contact mechanics seen with PCL deficiency [7, 21–25]. Changes seen during the gait cycle of PCL-deficient knees include a dynamic increase in tibiofemoral external rotation and varus [22]. In vivo biomechanical assessment of patients with complete isolated PCL tears compared to control patients show increased anteroposterior knee displacement, shear forces, and vertical ground reaction force during walking, with forces large enough to potentially damage the menisci and cartilage [7, 25]. These findings could help explain a greater risk of further intraarticular wear due to altered joint kinematics in patients with delayed multiligament PCL surgery.

Evidence is limited on the appropriate timing of surgical treatment for multiligament knee injury (MLKI) [3]. Prospective studies could ultimately provide the best evidence, but until then, retrospective data alongside biomechanical analyses suggest that MLKI with involvement of the PCL should often be treated acutely to prevent the incidence of additional intraarticular injuries, increase the treatability of meniscus tears, and potentially reduce the risk of early post-traumatic arthritis [26].

### Limitations

Several limitations to the current study should be considered. Selection bias was present in this retrospective study. A proportion of patients may have sustained severe injuries due to motor vehicle accident-related polytrauma, requiring clinical stabilization and optimization past 12 weeks prior to MLKI. Furthermore, surgeon decision on surgical timing likely depended on the pathology seen, making for an imperfect comparison between early and delayed groups. Patient-reported outcomes, radiographic findings, and clinical follow-up were outside the scope of the study, which focused on intraarticular surgical findings, but would provide more information on knee function following early versus delayed multiligament PCL-R. Observational bias of injury- and surgery-related variables are a further limitation of the study, as data was collected and documented based on surgical notes, rather than intraoperative images. Additionally, multiligament knee injuries are heterogeneous in their level of pathology, so study results and management considerations cannot apply to every such injury. In the absence of objective anteroposterior laxity measurements, the association between persistent tibiofemoral laxity and the increased prevalence of intraarticular injuries in the delayed multiligament PCL-R group cannot explicitly be stated. While the inclusion of 148 patients with MLKI in the current study should provide a reliable representation the concurrent patterns of injury and treatment in early and delayed multiligament PCL-R, future multicenter investigations and registry studies could provide a larger sample size.

## Conclusion

Patients undergoing delayed (> 12 weeks) multiligament PCL-R demonstrated a higher rate of chondral pathology, specifically of the lateral and medial femoral condyles, when compared to patients undergoing early PCL-R (≤ 12 weeks). Given higher rates of chondral pathology and medial meniscus surgery seen in delayed multiligament PCL-R, early management of PCL-based multiligament knee injury is recommended to restore knee stability and potentially prevent the development of further intraarticular injury.

## Data Availability

The datasets analyzed during the current study are available from the responsible institution on reasonable request.
